# Structure and regulation of ZCCHC4 in m^6^A-methylation of 28S rRNA

**DOI:** 10.1038/s41467-019-12923-x

**Published:** 2019-11-06

**Authors:** Wendan Ren, Jiuwei Lu, Mengjiang Huang, Linfeng Gao, Dongxu Li, Gang Greg Wang, Jikui Song

**Affiliations:** 1 Department of Biochemistry, University of California, Riverside, CA 92521 USA; 2 Environmental Toxicology Graduate Program, University of California, Riverside, CA 92521 USA; 3 Lineberger Comprehensive Cancer Center, University of North Carolina at Chapel Hill School of Medicine, Chapel Hill, NC 27599 USA; 4 Department of Biochemistry and Biophysics, University of North Carolina at Chapel Hill, Chapel Hill, NC 27599 USA

**Keywords:** Transferases, X-ray crystallography

## Abstract

*N*^6^-methyladenosine (m^6^A) modification provides an important epitranscriptomic mechanism that critically regulates RNA metabolism and function. However, how m^6^A writers attain substrate specificities remains unclear. We report the 3.1 Å-resolution crystal structure of human CCHC zinc finger-containing protein ZCCHC4, a 28S rRNA-specific m^6^A methyltransferase, bound to *S*-adenosyl-L-homocysteine. The methyltransferase (MTase) domain of ZCCHC4 is packed against N-terminal GRF-type and C2H2 zinc finger domains and a C-terminal CCHC domain, creating an integrated RNA-binding surface. Strikingly, the MTase domain adopts an autoinhibitory conformation, with a self-occluded catalytic site and a fully-closed cofactor pocket. Mutational and enzymatic analyses further substantiate the molecular basis for ZCCHC4-RNA recognition and a role of the stem-loop structure within substrate in governing the substrate specificity. Overall, this study unveils unique structural and enzymatic characteristics of ZCCHC4, distinctive from what was seen with the METTL family of m^6^A writers, providing the mechanistic basis for ZCCHC4 modulation of m^6^A RNA methylation.

## Abstract (web summary)

The N6-methyladenosine (m^6^A) RNA modification is an evolutionarily conserved epitranscriptomic mechanism that impacts several cellular processes. Here the authors present a structure-function analysis of the ZCCHC4, 28S RNA-specific m^6^A methyltransferase, shedding light on its regulation and mechanisms that ensure substrate specificity.

## Introduction

Covalent modifications of RNA molecules represent an evolutionarily conserved epitranscriptomic mechanism that critically regulates diverse cellular activities^1^. One of the most prevalent RNA modifications is *N*^6^-methyladenosine (m^6^A), which is widely present in mRNA and long noncoding RNA (lncRNA)^2^, as well as in the ribosomal RNA (rRNA)^3,4^ and small nuclear RNA (snRNA)^5,6^. Specific methylation of these RNA molecules functions to modulate RNA structure and protein–RNA interactions, which in turn influences RNA metabolism, cell signaling, cell survival, and differentiation^7,8^. Dysregulation of m^6^A-based RNA modification has been associated with severe human diseases, such as cancer^9,10^.

The dynamic profiling of m^6^A across the epitranscriptome is mediated by an array of distinct writer enzymes. For instance, mRNA and lncRNA are primarily methylated by the METTL3–METTL14 heterodimeric complex^11–15^, which recognizes a DRACH (D: A, G, U; R: G, A; H: A, C, U) consensus sequence near 3′ UTR^16–18^, whereas a subset of mRNA and snRNA sites is distinctly methylated by RNA methyltransferase METTL16^19^, which recognizes a UACAGAGAA motif embedded in a stem-loop structure^20,21^. In addition, m^6^A modification of cap adenosine at the transcription start nucleotide of mRNAs is achieved by cap-specific methyltransferase CAPAM, which then promotes translation of capped mRNAs^22^. Recent studies have further demonstrated that the m^6^A modification on the site 4220 of 28S rRNA and site 1832 of 18S rRNA is, respectively, mediated by CCHC zinc finger-containing protein ZCCHC4 and the methyltransferase METTL5^9,23^. Notably, these identified RNA methyltransferases bear no sequence homology, except for a remotely related methyltransferase (MTase) domain (Supplementary Fig. 1), raising a question of the molecular basis underlying their distinct substrate specificities.

ZCCHC4 is a newly identified m^6^A RNA methyltransferase and is highly conserved in multicellular organisms^9,23^. *ZCCHC4* knockout in human cells eliminates the m^6^A4220 methylation of 28S rRNA^9,23^, but not any other identified m^6^A sites^23^, suggesting its exclusive role in methylating 28S rRNA. ZCCHC4-mediated m^6^A4220 methylation in 28S rRNA promotes ribosome assembly and translation, which in turn impacts cell proliferation and tumor growth^9^. In addition, a previous study on K-Ras-transformed cells has identified ZCCHC4 as a potential factor required for Ras-mediated gene silencing^24^. However, due to the lack of structural information, the mechanism by which ZCCHC4 recognizes and methylates its RNA substrate remains unknown.

To provide mechanistic insights into the ZCCHC4-mediated m^6^A RNA methylation, we determined the crystal structure of human ZCCHC4 at 3.1 Å resolution. Distinct from what was observed for all other m^6^A methyltransferases, ZCCHC4 is organized into four domains, with three flanking zinc finger domains packed around the MTase domain, forming an integrated RNA-binding platform. Strikingly, the catalytic center is blocked by a linker sequence, namely regulatory loop, bridging the MTase domain and the C-terminal CCHC domain, resulting in an autoinhibitory conformation. Furthermore, the SAM-binding pocket adopts a fully closed conformation, suggesting of a mechanism by which cofactor binding is coupled with RNA substrate recognition. In addition, mutational and enzymatic analyses reveal a role of the stem-loop structure within the RNA substrate in governing the substrate specificity. Together, this work reveals the previously unappreciated molecular mechanisms that underlie activity autoregulation and substrate binding of ZCCHC4 in the modulation of cellular m^6^A RNA methylation.

## Results

### Overall structure of the ZCCHC4–SAH complex

**Fig. 1:**
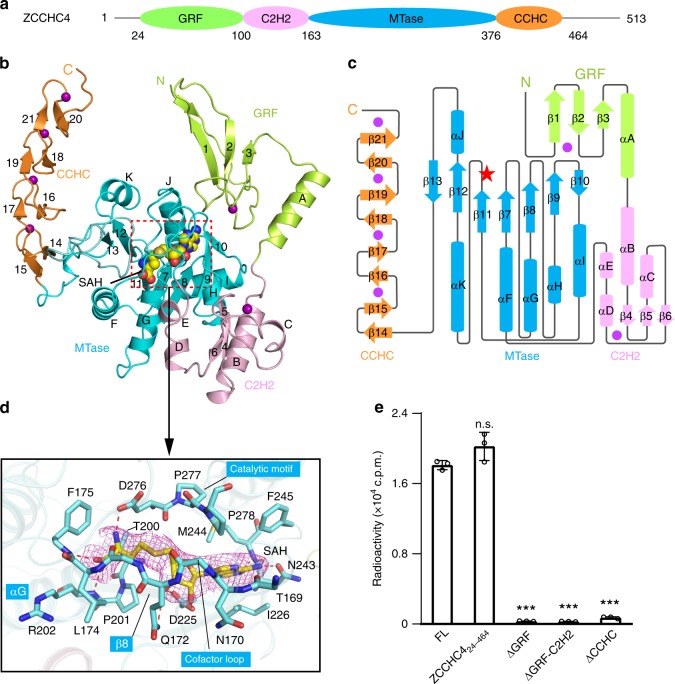
Crystal structure of ZCCHC4. **a** Domain architecture of ZCCHC4, with individual domains indicated by residue numbers. **b** Ribbon representation of ZCCHC4_24-464_ bound to SAH, with the β-strands and α-helices numbered numerically and alphabetically, respectively. The zinc ions are shown as purple spheres. **c** Schematic representation of the structure of ZCCHC4. The zinc ions are represented by purple spheres. The catalytic site is marked with red star. **d** Close-up view of the interaction between ZCCHC4 (cyan) and SAH (yellow), with hydrogen-bonding interactions depicted as dashed lines and the Fo–Fc omit map (magenta) of SAH contoured at 3σ level. **e** Methylation activity of full-length (FL), core (ZCCHC4_24-464_) or domain-truncated ZCCHC4. The data are mean ± SD. Statistical analysis used two-tailed Student’s *t* test for the difference from FL: ***p < 0.001. n.s., not significant. Source data are provided as a Source Data file

ZCCHC4 predictably contains a MTase domain with a DPPF catalytic motif, flanked by an N-terminal GRF (Gly–Arg–Phe)-type zinc finger domain and a C-terminal CCHC domain^9^ (Fig. 1a). Based on sequence conservation analysis (Supplementary Fig. 2a), we identified an evolutionarily conserved core fragment of human ZCCHC4 (residues 24–464; ZCCHC4_24-464_) (Fig. 1a, b), encompassing all the predicted domains. For successful crystallization, we further mutated five putative surface residues of ZCCHC4_24-464_ each into alanine (see the Methods section), which permitted crystallization and structure determination. The crystal structure of ZCCHC4_24-464_, in complex with *S*-adenosyl-L-homocysteine (SAH), product of methyl donor *S*-adenosyl-L-methionine (SAM), was refined to 3.1 Å resolution (Fig. 1b; Supplementary Table 1). There are six ZCCHC4_24-464_ complexes in one asymmetric unit; among these, two ZCCHC4 molecules engage swapping of the N-terminal tail (residues M26-V28) of one to pair in antiparallel with residues F48-V51 of the other (Supplementary Fig. 2b), likely due to crystal packing effects. These two molecules also yield the highest-quality electron density map, with one of which permitting the modeling of the entire segment of residues M25-G440. We therefore chose this ZCCHC4 molecule for structural analysis. The N-terminal regions of all the other ZCCHC4 molecules are not involved in domain swapping, in line with the fact that ZCCHC4 exists as a monomer in solution, as demonstrated by our size-exclusion chromatography analysis (Supplementary Fig. 2c).

The structure of ZCCHC4_24-464_ reveals four closely packed domains: an N-terminal GRF zinc finger domain is followed by a C2H2 zinc finger domain, an MTase domain, and a C-terminal CCHC zinc finger domain, folded into an integrated structural unit (Fig. 1b). The MTase domain is comprised of seven-stranded β-sheet, formed by five parallel strands and two antiparallel capping strands (Fig. 1b, c). This central β-sheet is further flanked by three intervening α-helices on each side (Fig. 1b). The SAH molecule snugly fits into a fully closed pocket, lined by a loop connecting the C2H2 domain and the MTase domain (cofactor loop: residues P163-F175), the terminal ends of αG-helix and β8-strand, and the DPPF (D276-P277-P278-F279) catalytic motif (Fig. 1d; Supplementary Fig. 3). The homocysteine moiety of SAH is surrounded by the side chains of L174-F175, T200-R202, and D276, with the amino group donating a hydrogen bond to the side-chain carboxylate of D276 and the carboxylate receiving a hydrogen bond from the backbone amide of F175 (Fig. 1d). On the other end, the adenosyl moiety of SAH is embraced by the side chains of I226, N243, M244, and F245 through van der Waals contacts, with the N6 atom further forming a hydrogen bond with the side-chain carbonyl group of N243 (Fig. 1d). In addition, the sugar moiety of SAH is recognized by Q172 and D225 through hydrogen-bonding interactions (Fig. 1d). It is worth noting that this closure of SAH-binding pocket is similar to that of CAPAM (Supplementary Fig. 4a, b), but distinct from those of METTL3, METTL5, and METTL16, in which the cofactor-binding pockets are rather exposed to the solvent (Supplementary Fig. 4c–e). In fact, the residues involved in the cofactor binding are poorly conserved among these m^6^A RNA methyltransferases (Supplementary Figs. 1, 4), highlighting their divergent cofactor-binding mechanisms.

The tandem GRF and C2H2 domains flank one end of the central β-sheet, with the GRF domain stacking on top of the SAH-binding pocket and the C2H2 domain docked in the groove formed by helices αG and αH of the MTase domain (Fig. 1b; Supplementary Fig. 5a). The structure of the GRF zinc finger domain is dominated by a three-stranded antiparallel β-sheet, with a CHCC zinc cluster embedded between the loop preceding β1 and the linker connecting β2 and β3 (Fig. 1b; Supplementary Fig. 5a, b). In addition, the three-stranded β-sheet of GRF is appended by a C-terminal α-helix that guides the packing of the GRF domain toward the MTase domain (Fig. 1b; Supplementary Fig. 5a). The C2H2 zinc finger domain adopts an α/β-fold arranged in an α-β-β-α-β-α-α fashion, with a C2H2 zinc cluster formed between the loop connecting β4 and β5 and the loop connecting β6 and αD (Fig. 1b; Supplementary Fig. 5a, c). Association of the GRF and C2H2 domains with the MTase domain is both mediated by surface complementarity, which permits formation of extensive interdomain van der Waals and hydrogen-bonding contacts centered around the SAH-binding pocket (Supplementary Fig. 5a). The opposite end of the central β-sheet is sided by the CCHC domain, comprised of four consecutive two-stranded β-sheets and a C-terminal tail, stacking on top of each other to form four CCHC zinc clusters in a spacing of 10–11 Å (Fig. 1b; Supplementary Fig. 5a, d). Association of the CCHC domain with the MTase domain is mainly mediated by hydrogen bonds formed between residues from the start strands of the CCHC domain and the C-terminal end of the MTase domain (Supplementary Fig. 5a). As a result of these domain arrangements, the GRF and CCHC domains both position one end to flank the catalytic site of the MTase domain, while extending the other end away from the MTase domain, each adopting a claw-like conformation roofing the catalytic site (Fig. 1b).

Structural homology search using the Dali server^25^ reveals that the GRF domain of ZCCHC4 is well aligned with the corresponding domain of APE2 (apurinic/apyrimidinic endonuclease 2) nuclease^26^ (Supplementary Fig. 5e); among the most conserved sites are those involved in the interactions of APE2 with single-stranded DNAs for 3′–5′ exonuclease processing^26^. The CCHC domain of ZCCHC4 bears a remote homology with the CCHC motif of the DHHC (Asp–His–His–Cys) palmitoyltransferases (Supplementary Fig. 5f), which harbors a catalytic center for protein palmitoylation^27^. On the other hand, there is no structural homologue identified for the C2H2 domain.

### The MTase-flanking domains are required for ZCCHC4 activity

The close association between MTase and the flanking domains potentially creates an extended RNA-binding surface, implying a possible contribution of the flanking domains to the RNA binding of ZCCHC4 (Fig. 1b). To test the role of the MTase-flanking domains in ZCCHC4-mediated RNA methylation, we have measured the methylation activity of ZCCHC4, either intact or domain-truncated, on a 30-mer RNA substrate, derived from the Ade4220-containing segment of 28S rRNA (Cyt4199-Gua4228) (Fig. 1e). As expected, the ZCCHC4_24-464_ core fragment shows a comparable activity with full-length ZCCHC4. In contrast, deletion of the C-terminal CCHC domain (ΔCCHC) led to a dramatic reduction of enzymatic activity, while deletion of the N-terminal GRF domain, either alone (ΔGRF) or together with the C2H2 domain (ΔGRF-C2H2), completely abolished the activity of ZCCHC4, indicating the critical requirement of these domains for ZCCHC4-mediated RNA methylation. Consistently, electrophoretic mobility shift assays (EMSA) revealed that, in comparison with wild-type ZCCHC4_24-464_, the ΔGRF, ΔGRF-C2H2, and ΔCCHC truncations all substantially impair the RNA-binding affinity of ZCCHC4 (Supplementary Fig. 5 g). Analysis of 1D ^1^H NMR spectra of these domain-truncated ZCCHC4 fragments indicate that they remain folded in solution (Supplementary Fig. 6), supporting a direct role of the flanking domains in mediating substrate binding of ZCCHC4.

### ZCCHC4 MTase domain adopts an autoinhibitory conformation

**Fig. 2:**
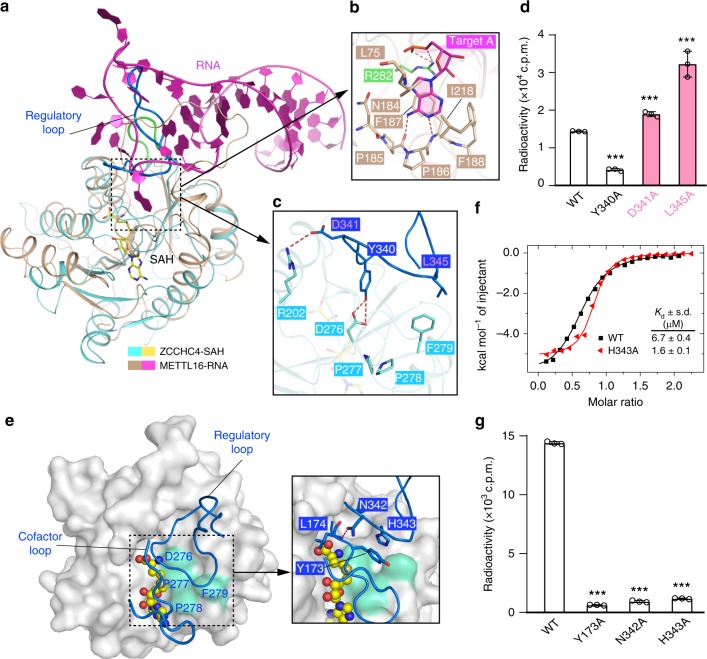
The regulatory loop controls the enzymatic activity of ZCCHC4. **a** Structural alignment of ZCCHC4 MTase domain (aquamarine) and HP1 RNA (magenta)-bound METTL16 (wheat) (PDB 6DU4 [https://www.rcsb.org/structure/6DU4]). The regulatory loop and its corresponding region in METTL16 are colored marine and green, respectively. The MTase-associated domains of ZCCHC4 were removed for clarity. **b** Close-up view of the interaction between METTL16 and the target adenosine. **c** Close-up view of the interaction between the regulatory loop and catalytic site residues. The hydrogen-bonding interactions are depicted as dashed lines. **d** Methylation activity of the regulatory loop mutants of ZCCHC4. The two activity-enhancing mutants are colored in hot pink. **e** Surface view of the ZCCHC4 MTase domain, with the interaction between the regulatory loop and cofactor loop (both colored marine) stabilizing the closed conformation of the SAH-binding pocket. The SAH molecule is shown in sphere representation. The surface of the catalytic motif is colored in green-cyan. **f** ITC binding curves of SAM over wild-type (WT) or H343A ZCCHC4. The *K*_d_ values and error estimates were derived from two independent measurements. **g** Methylation activity of the mutants on the regulatory loop-cofactor loop interface. Statistical analysis used two-tailed Student’s *t* test for the difference from WT: ***p < 0.001. Source data are provided as a Source Data file

The MTase domain of ZCCHC4 merely shares ~6–17% sequence identity with the corresponding domains of all other m^6^A RNA methyltransferases identified so far (Supplementary Fig. 1). Nevertheless, their structures exhibit a similar Rossmann fold, with the catalytic motif aligned next to the SAH-binding pocket (Fig. 2a–c; Supplementary Fig. 7a–d), suggestive of a conserved catalytic mechanism. Astonishingly, structural comparison of ZCCHC4 with METTL16, as well as other RNA methyltransferases, also reveals a striking difference in the active-site conformation: the D/N-P-P-F/W catalytic motifs of METTL16, METTL3, METTL5, and CAPAM are all readily accessible for RNA contacts (Fig. 2b; Supplementary Fig. 7a–d); in contrast, the corresponding DPPF motif in ZCCHC4 is shielded from solvent exposure by the loop connecting αK and β13 (hereafter referred to as regulatory loop), which wedges into the catalytic center for interaction with the active-site residues (Fig. 2a, c). Of particular note, residue D276 forms a bifurcated side-chain hydrogen bond with regulatory loop residue Y340 (Fig. 2c), which potentially occludes D276 from nucleophilic attack during the methylation reaction. Furthermore, the aromatic ring of residue F279, which presumably aligns the target base during the methylation reaction (Fig. 2b), stacks against the aliphatic chain of regulatory loop residue L345 (Fig. 2c). In addition, regulatory loop residue D341 reaches over to form a salt bridge with R202 on the SAH-binding pocket, which further supports the positioning of the regulatory loop to the active site. These observations suggest that ZCCHC4 adopts an autoinhibitory conformation.

To test the effect of the intramolecular interactions observed above on the methylation activity of ZCCHC4, we have selected the active site-contacting residues, including Y340, D341, and L345, of the regulatory loop for mutagenesis and enzymatic assay. In comparison with wild-type ZCCHC4, mutation of residues D341 and L345 each into alanine led to a 1.3- and 2.2-fold increase in RNA methylation (Fig. 2d), supporting the idea that the intramolecular interaction between the regulatory loop and the catalytic motif results in enzymatic inhibition of ZCCHC4. On the other hand, introduction of the Y340A mutation severely impairs the methylation activity of ZCCHC4, suggesting that residue Y340 might also play an additional role in the enzymatic catalysis. Indeed, structural alignment of ZCCHC4 and the RNA-bound METTL16 reveals that in METTL16, the segment that corresponds to the regulatory loop engages extensive contacts with the RNA loop (Fig. 2a, b), implying a similar role of the regulatory loop of ZCCHC4 in substrate recognition, and a conformational readjustment of the regulatory loop accompanying this process. Together, these data suggest that ZCCHC4 adopts an autoinhibitory conformation that may regulate its RNA substrate specificity.

### Regulation of ZCCHC4 on SAM binding and RNA methylation

Structural analysis of ZCCHC4 further reveals an interaction between the regulatory loop and the cofactor loop (Fig. 2e): residues N342 and H343 from the regulatory loop interact with residues L174 and Y173 on the cofactor loop through hydrogen-bonding and ring-stacking interactions, respectively (Fig. 2e). These interactions presumably stabilize the closed conformation of the SAM-binding pocket in the RNA-free state of ZCCHC4, therefore implying a role of the regulatory loop in controlling the cofactor binding of ZCCHC4. To test this possibility, we measured the SAM-binding affinities of wild-type and H343A-mutated ZCCHC4 by Isothermal Titration Calorimetry (ITC). Wild-type ZCCHC binds to SAM with a dissociation constant (*K*_d_) of 6.7 μM (Fig. 2f; Supplementary Fig. 7e), consistent with the extensive interactions between ZCCHC4 and SAH (Fig. 1c). In contrast, titration of the H343A mutant with SAM gave a *K*_d_ of 1.6 μM (Fig. 2f; Supplementary Fig. 7f), ~four fold stronger than the binding between wild-type ZCCHC4 and SAM, indicating that disruption of the intramolecular interaction between the regulatory loop and cofactor loop significantly increases the SAM-binding affinity of ZCCHC4.

To explore the role of the cofactor loop in the ZCCHC4-mediated RNA methylation, we modeled the target adenosine into the active site of ZCCHC4 based on structural alignment of the ZCCHC4 MTase domain and HP1 RNA-bound METTL16 (PDB 6DU4)^20^ (Supplementary Fig. 7g). The structural model indicates that the cofactor loop is in close proximity to the RNA substrate, with loop residue Y173 flanking the adenosine ring in a fashion similar to that of residues L75 and I218 of METTL16 (Fig. 2b; Supplementary Fig. 7g). Consistently, mutation of cofactor loop residue Y173 into alanine largely abolishes the methylation activity of ZCCHC4 (Fig. 2g). Likewise, mutations of the regulatory loop residues N342 and H343 each into alanine also severely impair the activity of ZCCHC4 (Fig. 2g), reinforcing the notion that the regulatory loop is engaged in the ZCCHC4-mediated methylation. Note that EMSA assays indicated that the mutations on the regulatory loop do not significantly affect the RNA-binding activity of ZCCHC4 (Supplementary Fig. 8a), suggesting a role of these residues in enzymatic catalysis, rather than substrate binding of ZCCHC4. Together, these studies suggest that the intramolecular interaction between the regulatory loop and the cofactor loop might not only restrict the SAM-binding activity of ZCCHC4 but also strengthen the autoinhibitory regulation mediated by the regulatory loop, thereby establishing a link between the RNA recognition of ZCCHC4 and its SAM-binding activity.

### Sequence- and structure-recognition of 28S rRNA by ZCCHC4

**Fig. 3:**
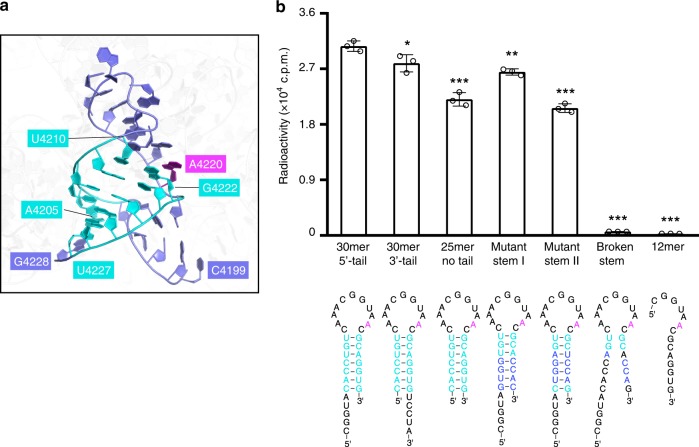
Substrate specificity of ZCCHC4. **a** The cryo-EM structure of 28S rRNA (PDB 4UG0 [https://www.rcsb.org/structure/4UG0]) highlighting the local conformation of the 30-mer RNA substrate used in this study. The stem nucleotides are colored cyan. Target Ade4220 and the rest of 30-mer RNA are colored magenta and slate, respectively. Other regions of the 28S rRNA are colored gray in transparent view. **b** Methylation activity of ZCCHC4 on the indicated RNA substrates, with the corresponding schematics shown beneath. The data are mean ± SD. Statistical analysis used two-tailed Student’s *t* test for the difference from native 30-mer RNA with 5′-tail: *p < 0.05; **p < 0.01; ***p < 0.001. Source data are provided as a Source Data file

Previous studies have indicated that ZCCHC4, with substrate preference toward the AAC RNA motif in vitro, is highly specific toward Ade4220 of 28S rRNA^9,23^, located on the helix H81^28,29^. Structural analysis of the cryo-EM structure of human 80S ribosome^30^ reveals that the Ade4220-residing segment adopts a stem-loop structure, with nucleotides Cyt4211-Cyt4221 extending as a twisted loop on top of a six base pair (bp)-long stem (Fig. 3a). To determine whether the RNA secondary structure contributes to the substrate specificity of ZCCHC4, we compared the methylation activity of ZCCHC4 on the 30-mer RNA (Cyt4199-Gua4228), encompassing the stem-loop segment as well as a 5 nucleotide (nt)-long 5′ overhang, and a 12-mer RNA (Cyt4215-Gua4226) that was used by a previous study^9^, which presumably lacks of any secondary structure (Fig. 3b). ZCCHC4 shows a 104-fold higher activity on the 30-mer RNA over the 12-mer RNA (Fig. 3b), suggesting that the structural feature of the 30-mer RNA may influence the methylation efficiency of ZCCHC4. Next, we aimed to disrupt the RNA stem structure by mutating four stem nucleotides, Ura4208, Gua4225, Gua4226, and Ura4227, into Ade4208, Cyt4225, Cyt4226, and Ade4227, respectably. The methylation activity of ZCCHC4 shows a 50-fold reduction on the “broken stem” RNA than on the native 28S RNA (Fig. 3b). In contrast, swapping of four complementary bases on the stem that are either 2- or 3-bp away from the RNA loop, only led to 1.2- and 1.5-fold reduction of the enzymatic activity, respectively (Fig. 3b). Similarly, removal of the 5′ overhang or retaining a same-length 3′ overhang instead only led to a modest decrease of the methylation activity of ZCCHC4, suggesting that the stem, rather than the overhang sequence, is essential for the substrate specificity of ZCCHC4. Together, these observations suggest that ZCCHC4 recognizes a combined sequence and structural feature of 28S rRNA.

### Mapping of the RNA-binding surface of ZCCHC4

**Fig. 4:**
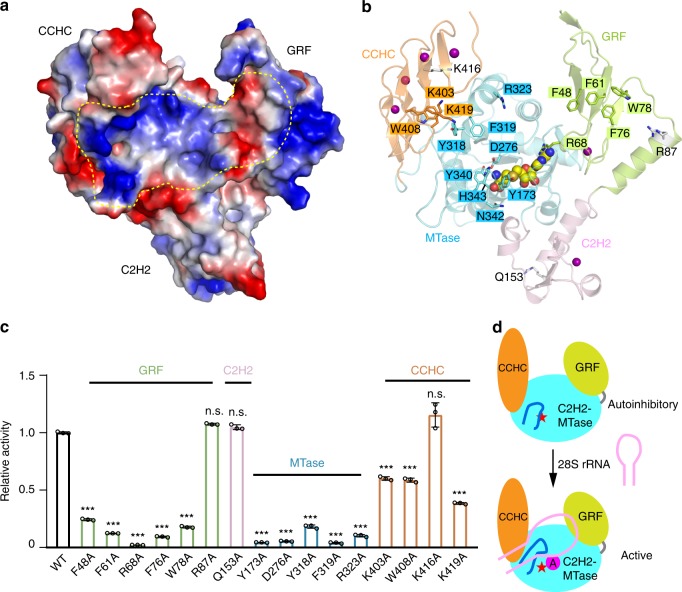
Mapping of the substrate-recognition surface of ZCCHC4. **a** Electrostatic surface of ZCCHC4_24-464_, with the potential RNA-binding surface marked by dashed circle. **b** The mutational sites associated with the activity loss of ZCCHC4 are shown in stick representation and color-coded in the same fashion as the corresponding domains. The residues whose mutations failed to affect ZCCHC4 activity appreciably are shown as gray sticks. **c** Relative methylation activity of selected ZCCHC4_24-513_ mutants over WT. The data are mean ± SD. Statistical analysis used two-tailed Student’s t test for the difference from WT: n.s., not significant; ***p < 0.001. Source data are provided as a Source Data file. **d** Model for the substrate recognition of ZCCHC4. The regulatory loop (blue) occludes the active site (red star) in RNA-free state. In the presence of the cognate substrate 28S rRNA, the regulatory loop readjusts conformation for RNA interaction, leading to the active state of ZCCHC4

Structural analysis of ZCCHC4, along with the aforementioned RNA binding and enzymatic analyses, argues for an important role of the MTase-associated domains in ZCCHC4-mediated RNA methylation. Analysis of the electrostatic surface of ZCCHC4 further reveals that the GRF domain forms a hydrophobic patch next to the active site (Fig. 4a, b), which might serve as an extended RNA-binding surface. In support of this notion, residues F61, R68, F76, and W78 on the GRF domain are evolutionarily conserved, with F61 representing one of the signature residues in the GRF domain-defining motif (Supplementary Figs. 1, 5e). In fact, a previous study, based on NMR chemical shift perturbation analysis, has demonstrated that the counterparts of these residues in the GRF domain of APE2 mediate the binding of APE2 to the single-stranded DNA^26^. On the opposite side of the catalytic center, an interdomain cleft formed between the MTase and CCHC domains presents another surface patch containing basic and hydrophobic residues (Fig. 4a, b), which potentially also contributes to the substrate binding of ZCCHC4.

To further analyze the RNA-binding sites of ZCCHC4, we have selected a number of residues from these surface patches of the GRF and CCHC domains, along with the residues surrounding the catalytic site of the MTase domain, for mutagenesis and enzymatic assays. Our results showed that mutation of residues on the surface patches of the GRF and MTase domains severely compromised the activity of ZCCHC4 (Fig. 4c). Mutations of the residues on the surface patch in the interdomain cleft between the MTase and CCHC4 domains also significantly reduced the activity of ZCCHC4, albeit to a lesser extent (Fig. 4c). Note that none of these mutations led to a substantial decrease in RNA-binding activity of ZCCHC4 (Supplementary Fig. 8b), highlighting the delicacy of RNA substrate binding in ZCCHC4-mediated methylation. The mutational effects of these residues are well in line with their sequence conservation (Supplementary Fig. 8c), indicating their important roles in ZCCHC4-mediated RNA methylation. In contrast, mutation of residue K419 located further away from the MTase domain, residue Q153 on the C2H2 domain, or residue R87 on the GRF domain, did not appreciably affect the enzymatic activity of ZCCHC4 (Fig. 4c), which sets a boundary for the potential RNA-binding surface of ZCCHC4. It is worth noting that, out of the five surface mutations introduced for crystallization, sites K167 and K168 both fall into this RNA-binding surface of ZCCHC4, consistent with the observation for a large reduction of ZCCHC4 activity by these mutations (Supplementary Fig. 8d, e). Together, these mutational analyses provide a sketched view on the RNA-binding surface of ZCCHC4, spanning from the GRF domain to the CCHC domain.

## Discussion

The m^6^A RNA methylation is increasingly appreciated as an important epitranscriptomic mechanism for regulation of the metabolism, trafficking and/or function of a diverse array of RNA molecules, which critically impacts on the behavior and phenotype of cells. To date, little is known about how different m^6^A writer enzymes target distinct RNA substrates for carrying out their respective roles in RNA modulation. Through structural, biochemical, and enzymatic characterization of ZCCHC4, a recently reported rRNA m^6^A writer, this study reveals the molecular basis for the ZCCHC4-mediated m^6^A4220 modification of 28S rRNA. Importantly, ZCCHC4 forms a multidomain RNA-binding platform, permitting its specific recognition of a stem-loop feature inherent in the 28S rRNA substrate. Furthermore, it reveals a loop segment that controls both the enzymatic catalysis of ZCCHC4 and its SAM-binding activity, providing a potential mechanism by which ZCCHC4 achieves substrate specificity. This study therefore illustrates a previously unappreciated dynamic interplay between the RNA substrate and its m^6^A writer, with important implication in the functional regulation of ribosome assembly, translation, and cell proliferation.

Uniquely among all the m^6^A RNA methyltransferases identified so far, ZCCHC4 manifests an autoinhibitory conformation, with the regulatory loop occluding the catalytic site from RNA binding. Our structural modeling analysis, along with enzymatic assays, demonstrated that the regulatory loop may serve as a key element that couples RNA substrate recognition with the conformational state of ZCCHC4. Although the functional implication of this intramolecular regulation remains to be determined, it is conceivable that the interaction between the regulatory loop and the 28S rRNA leads to conformational rearrangement of ZCCHC4, thereby switching ZCCHC4 from an inactive state to an enzymatically active state (Fig. 4d). The other important observation is that the SAH-binding pocket adopts a fully closed conformation, which is in part strengthened by the intramolecular interaction between the regulatory loop and cofactor loop. It is therefore likely that the RNA substrate binding-triggered conformational change of the regulatory loop may also lead to destabilization of this intramolecular interaction, which consequently enhances the SAM-binding affinity of ZCCHC4. Together, these intramolecular interactions of the regulatory loop likely provide a gating mechanism for the RNA substrate and cofactor binding of ZCCHC4, which may contribute to its substrate specificity. This regulatory mechanism is reminiscent of mammalian DNA methyltransferase 1 (DNMT1)-mediated DNA methylation, in which an interdomain linker of DNMT1 mediates an autoinhibitory conformation to control its substrate specificity^31^. It is worth mentioning that an autoinhibitory conformation was also previously observed for METTL16, but likely in a different functional context^20^: the RNA binding of METTL16 triggers a conformational re-organization of the SAM-binding pocket (Supplementary Fig. 8 f), which in turn reduces the activity of METTL16^20^. Likewise, binding of SAM or SAH to METTL3 also induces a conformational change of a loop segment at the cofactor-binding site, namely gate loop, resulting in binding-induced folding of the SAM-binding pocket (Supplementary Fig. 8g)^13,14^. These observations suggest that cofactor binding-coupled conformational changes may represent a common mechanism for various m^6^A writers.

Previous studies have demonstrated that ZCCHC4 shows high specificity toward 28S rRNA: it specifically methylates Ade4220 of 28S rRNA, but not Ade1832 of 18S rRNA, the other methylation site of rRNA, even though both sites are embedded in an AAC motif^9^. Through nucleotide complementation analysis, this study demonstrates that the stem-loop structure critically influences the methylation activity of ZCCHC4. In this regard, structural analysis of 18S rRNA reveals a discrete structural environment for site 1832, which forms a base pair with Cyt1732 at the base of helix 44 (Supplementary Fig. 8h), providing an explanation for the lack of methylation activity of ZCCHC4 on this site. Through nucleotide replacement analysis, this study suggests that the stem-loop structural feature of 28S rRNA significantly contributes to the substrate specificity of ZCCHC4. Future investigation is warranted to further determine whether or not the stem region of the RNA substrate directly engages ZCCHC4 interaction or functions to stabilize the conformation of the RNA loop.

Lastly, this study has demonstrated how various structural modules within ZCCHC4 act in synergy for mediating the binding and methylation of substrates. In particular, the GRF and CCHC modules, through direct association with the MTase domain, extend the substrate-binding surface, permitting a rather large RNA-binding platform to accommodate the substrate specificity. In fact, all the RNA m^6^A methyltransferases identified so far, except for METTL5, contain MTase-flanking domains, suggesting a flanking-module-directed regulation, as we herein show for ZCCHC4, as a widely adopted mechanism for m^6^A writers, ensuring both binding affinity and substrate specificity. For instance, METTL3 harbors an N-terminal CCCH zinc finger domain that specifically recognizes the RNA substrate containing the GGACU motif^32^, which is essential for the methylation activity of the METTL3–METTL14 complex^14,32^. The detailed mechanism by which the MTase-associated domains of ZCCHC4 regulate its activity awaits further investigation.

## Methods

### Protein expression and purification

DNAs encoding human ZCCHC4, full-length or various fragments (ZCCHC4_24-513_: residues 24-513; ZCCHC4_24-464_: residues 24–464; ΔCCHC: residues 24–368; ΔGRF: residues 100–464; ΔGRF-C2H2: residues 159–464) were each inserted into a modified pRSF-Duet vector, preceded by an N-terminal hexahistidine (His_6_)-SUMO tag and a ubiquitin-like protease 1 (ULP1) cleavage site. To promote crystallization, five surface mutations (K55A, E56A, E57A, K167A, and K168A) were introduced onto the ZCCHC4_24-464_ construct, which presumably would lead to surface entropy reduction. On the other hand, for biochemical and enzymatic characterizations, various point mutations were generated based on the wild-type ZCCHC4_24-513_ construct. For protein expression, BL21(DE3) RIL cells harboring the expression plasmids were induced by addition of 0.4 mM isopropyl-β-D-thiogalactoside (IPTG) when the cell density reached optical density at 600 nm (A_600_) of 0.8, and continued to grow at 15 °C overnight. The cells were harvested and lysed in buffer containing 50 mM Tris–HCl (pH 8.0), 1 M NaCl, and 25 mM imidazole, 10% glycerol, 10 µg/mL DNase I, 10 µg/mL RNase A, and 1 mM PMSF. Subsequently, the fusion proteins were purified through a nickel column, followed by removal of His_6_-SUMO tag by ULP1 cleavage, ion-exchange chromatography and size-exclusion chromatography. The purified protein samples were concentrated in 20 mM Tris–HCl (pH 7.5), 100 mM NaCl, 5% glycerol, and 5 mM DTT, and stored at −80 °C freezer before use.

### Crystallization and structure determination

For crystallization of the ZCCHC4–SAH complex, 10 mg/mL ZCCHC4 was mixed with SAH in a molar ratio of 1:5, before incubation with 0.03 M citric acid, 0.07 M Bis–Tris propane (pH 7.6), 11–12% PEG3350, 10% glycerol, and 5 mM TCEP (Tris (2-carboxyethyl) phosphine hydrochloride) (pH 7.0) using the sitting drop vapor-diffusion method at 12 °C. For crystal harvesting, the crystals were soaked in the well solution supplemented with 20% (v/v) ethylene glycol before flash frozen in liquid nitrogen. X-ray diffraction data were collected on beamline 5.0.2 at the Advanced Light Source (ALS), Lawrence Berkeley National Laboratory with a wavelength of 1.283 Å and processed with the HKL3000 program^33^. Experimental phasing and initial model building was carried out by the Zn-SAD method using the CRANK2 program^34^. Subsequently, iterative cycles of model building and refinement were carried out using COOT^35^ and PHENIX^36^, respectively. The statistics for data processing and structure refinement are summarized in Supplementary Table 1.

### Electrophoretic mobility shift assay

To measure the binding between ZCCHC4 and the 30-mer 28S rRNA (5′-CGGUACA CCUGUCAAACGGUAACGCAGGUG-3′), unless indicated otherwise, 0.3 μM RNA was incubated with 0, 0.3, 0.6, or 1.2 μM ZCCHC4_24-513_ in buffer containing 20 mM Tris–HCl (pH 7.5), 100 mM NaCl, 5% glycerol, 1 mM DTT at 4 °C for 1 h. Five μL of the mixture was loaded and resolved in 6% TBE native gel at 4 °C under 100 V for 0.5 h. The gel image was visualized by SYBR Gold (Thermo Fisher) staining and subsequently by Coomassie blue staining.

### RNA methylation kinetics assay

The RNA methylation assays follow a previously published protocol (Song et al.^31^) with modifications. Each reaction mixture contains 25 nM ZCCHC4_24-513_, wild-type or mutants, 0.5 μM *S*-adenosyl-L-[^3^H] methionine (Perkin Elmer) and 1 μM RNA substrate in 50 mM Tris–HCl (pH 8.0), 7 mM β-ME, 5% glycerol, 100 μg/mL BSA, and 10 μM zinc acetate. The reaction mixture was incubated at 37 °C for 15–20 min, before quenched by 2 mM cold SAM. Ten μL of the reaction mixture was applied onto DEAE filtermat (Perkin Elmer), sequentially washed with 0.2 M ammonium bicarbonate (pH 8.2) twice, water once, and ethanol once. The filter paper was then air dried and soaked in ScintiVerse cocktail (Thermo fisher). The activity was measured by Beckman LS6500 scintillation counter. All the experiments were performed in three biological replicates and repeated with consistent results.

### ITC measurements

ITC measurements were performed using a MicroCal iTC200 instrument (GE Healthcare). To measure the bindings between the ZCCHC4_24-513_, WT or H343A mutant, and SAM, 0.1 mM ZCCHC4_24-513_ protein sample was dialyzed against buffer (20 mM Tris–HCl (pH 7.5), 100 mM NaCl, 1 mM TCEP) overnight. 1 mM SAM compound (Sigma) was dissolved in the same buffer. Titration of SAM in the syringe over ZCCHC4_24-513_ samples was carried out at 5 °C. The time between each injection was set at 180 s. All data were processed with the MicroCal Origin software and fitted with the single-site binding mode. The *K*_d_ values and error estimates were derived from the duplicated experiments.

### NMR experiments

For ^1^H NMR experiments, 0.05 mM purified mutant ZCCHC4 proteins were dissolved in 250 μL buffer containing 50 mM sodium phosphate (pH 6.7), 1–3 mM Tris–HCl, 150 mM NaCl, and 10% D_2_O. NMR spectra were collected on a Bruker Advance 700 MHz NMR spectrometer equipped with a TXI probe at 25 °C and processed using the Bruker TopSpin software.

### Reporting summary

Further information on research design is available in the Nature Research Reporting Summary linked to this article.

## Supplementary information

Supplementary Information

Reporting Summary

Source Data

## Data Availability

Coordinates and structure factors for ZCCHC4–SAH complex have been deposited in the Protein Data Bank under accession code 6UCA. The PDB accession codes 4UG0, 5K7M, 5K7W, 5L6D, 5IL2, 6IRY, 6DU4, 6B92, 6BML, and 6H2U were used in this study. All other data are available from the corresponding authors upon reasonable request. The source data underlying Figs. 1e, 2d, g, 3b, and 4c, and Supplementary Figs. 5g and 8a, b, e are provided as a Source Data file.
