# Supplementary material for: Structure and regulation of ZCCHC4 in m6A-methylation of 28S rRNA
Source: Nat Commun. 2019 Nov 6;10:5042. doi: 10.1038/s41467-019-12923-x (PMC6834594; doi:10.1038/s41467-019-12923-x)
Supplement: Supplementary file 1 — Supplementary Information [file 41467_2019_12923_MOESM1_ESM.pdf]

Supplementary Information

“Structure and regulation of ZCCHC4 in m^{6}A-methylation of 28S rRNA”

Ren et al.

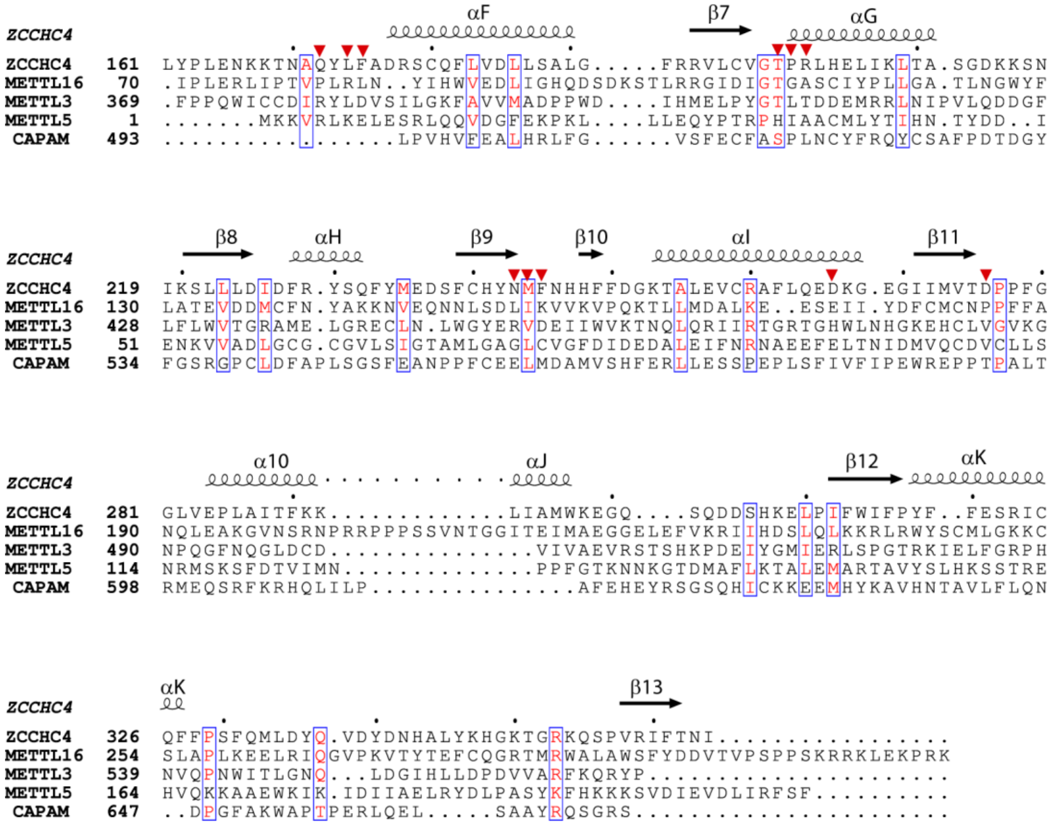

Supplementary Figure 1. Sequence alignment of the MTase domains of ZCCHC4 and other identified m^{6}A RNA methyltransferases. The secondary structures of ZCCHC4 MTase domain are indicated on top, with the corresponding numbering following the nomenclature in Fig. 1b. Similar residues are colored in red. The SAH-binding residues of ZCCHC4 are marked by red arrows on top.

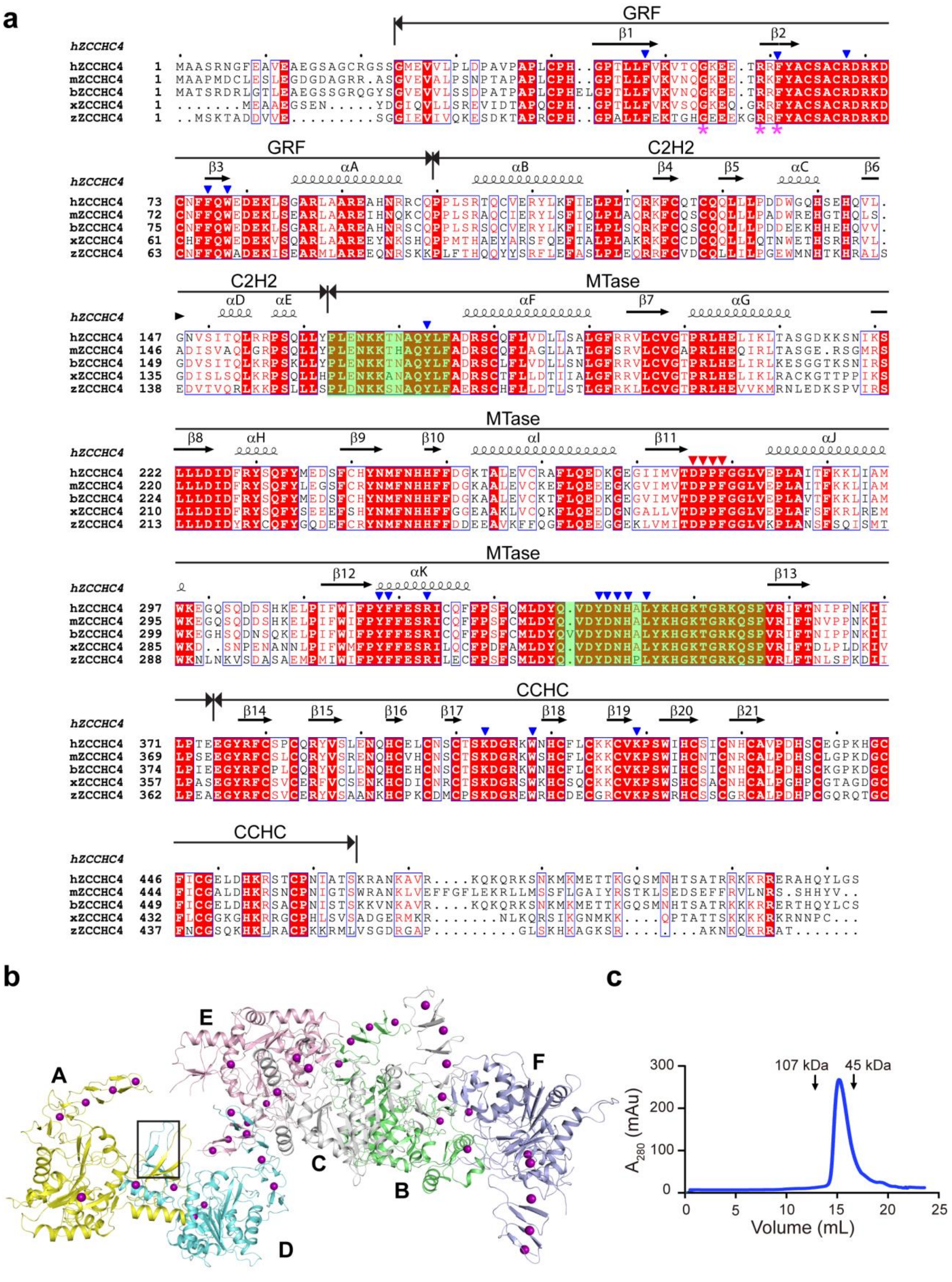

a
GRF
C2H2
MTase
CCHC
hZCCHC4
mZCCHC4
bZCCHC4
xZCCHC4
zZCCHC4
b
A
B
C
D
E
F
c
300
200
100
0
5
10
15
20
25
107 kDa
45 kDa
A280 (mAu)
Volume (mL)

Supplementary Figure 2. Sequence and structural analysis of ZCCHC4. (a) Sequence alignment of ZCCHC4 from human (hZCCHC4), mouse (mZCCHC4), bovine (bZCCHC4), Zebrafish (zZCCHC4) and Xenopus (xZCCHC4), with the individual domains and secondary structures of hZCCHC4 indicated on top. Although only the vertebrate orthologues are selected for comparison, ZCCHC4 is conserved in metazoans. Identical residues are colored white in red background. Similar residues are colored red. The cofactor-binding loop and regulatory loop are shaded green. The residues required for the activity of ZCCHC4 are marked by blue arrows. The DPPF catalytic motif is marked by red arrows. The three signature residues of the GRF domain, G54-R59-F61, are marked by magenta asterisks beneath. (b) Crystal structures of the six color-coded $ZCCHC4_{24\text{-}464}$ molecules in one asymmetric unit. The N-terminal tails of two ZCCHC4 molecules, which undergo domain swapping, are boxed. One of the ZCCHC4 molecules (aquamarine) involved in the domain swapping yields the best model-to-map fit, therefore has been selected for structural analysis. The chain IDs for individual molecules are labeled. (c) Sample elution profile of full-length ZCCHC4 on a Superdex 10/300 increase column. The elution volumes corresponding to proteins with molecular weight of 45 kDa and 107 kDa are marked. The molecular weight for monomeric ZCCHC4 is 59 kDa.

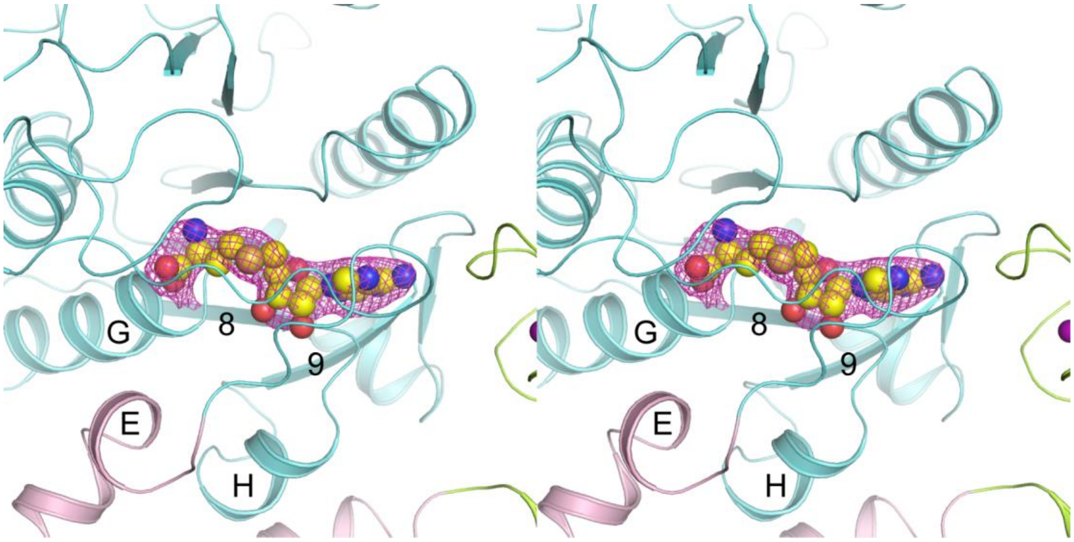

Supplementary Figure 3. Stereo view of the SAH-binding pocket in ZCCHC4. Fo-Fc omit map for SAH (sphere representation) is displayed as a magenta mesh, contoured at 3.0 σ level. The SAH-surrounding secondary structures are labeled.

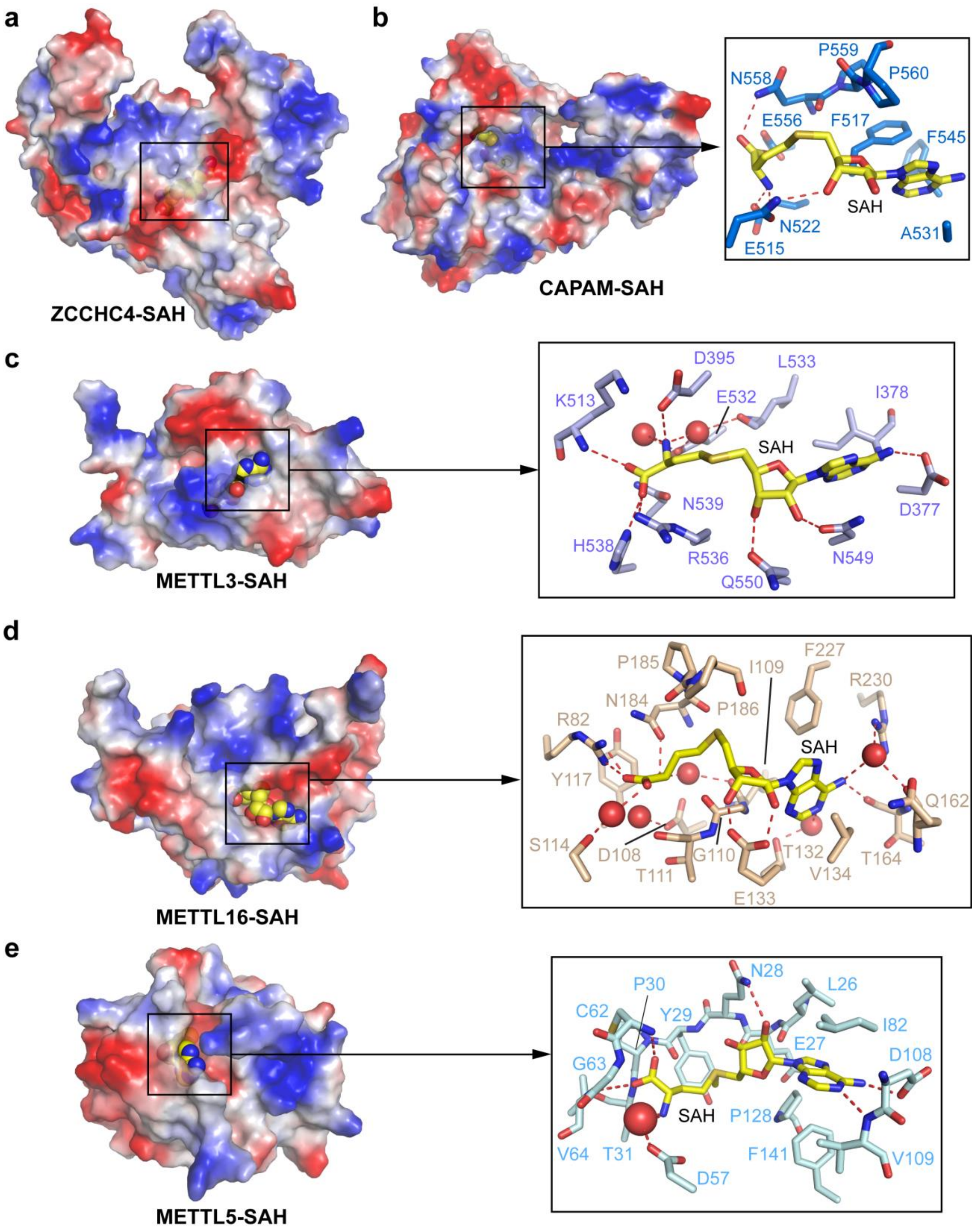

Supplementary Figure 4. Structural comparison of the cofactor-binding pockets of m^{6}A methyltransferases. (a) Electrostatic surface of ZCCHC4$_{24\text{-}464}$-SAH complex,

with a fully closed pocket (boxed) burying the SAH compound. (b) Electrostatic surface of CAPAM-SAH complex (PDB 6IRY)[1], with the SAH-binding pocket shown in the expanded view. (c) Electrostatic surface of METTL3-SAH complex (PDB 5L6D)[2], with the SAH-binding pocket shown in the expanded view. (d) Electrostatic surface of METTL16-SAH complex (PDB 6B92)[3], with the SAH-binding pocket shown in the expanded view. (e) Electrostatic surface of METTL5-SAH complex (PDB 6H2U)[4], with the SAH-binding pocket shown in the expanded view. The SAH is shown in sphere representation on the left panel (a-e). The protein residues and SAH compound are shown in stick representation in the expanded views. The hydrogen bonding interactions are shown as dashed lines.

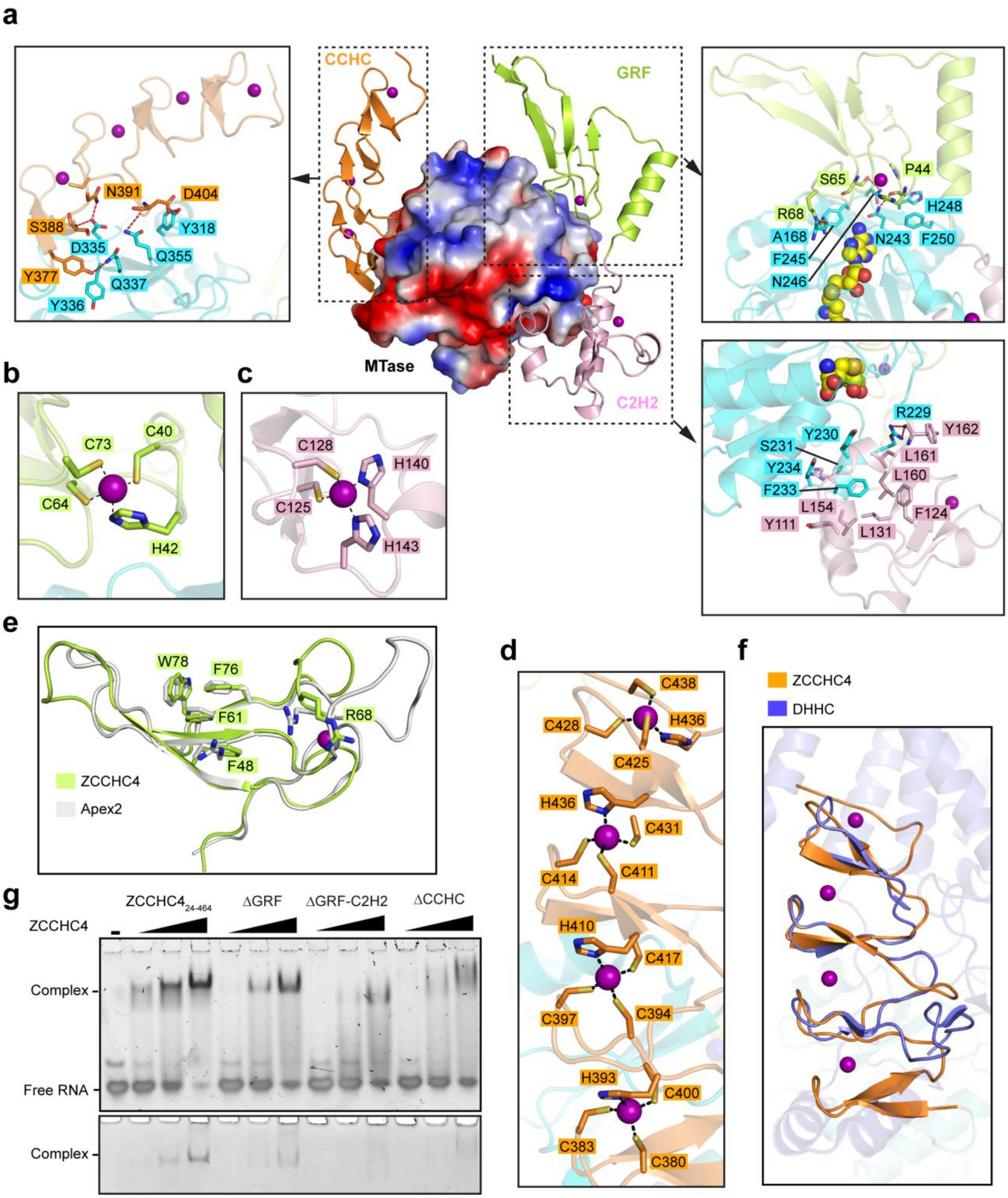

Supplementary Figure 5. Structural and biochemical analysis of ZCCHC4 domains. (a) The structure of ZCCHC4$_{24\text{-}464}$ with ribbon representations of the GRF, C2H2 and CCHC domains and electrostatic surface of the MTase domain. The residues involved in interdomain contacts are shown in the expanded views. The hydrogen bonds are depicted as dashed lines. (b) Close-up view of the zinc cluster formed in the

GRF domain. (c) Close-up view of the zinc cluster formed in the C2H2 domain. (d) Close-up view of the zinc clusters formed in the CCHC domain. (e) Structural alignment of the GRF domains of ZCCHC4 (limon) and APE2 (grey; PDB 5U6Z), with the side chains of the conserved residues shown in stick representation. Residue F48 of ZCCHC4, which corresponds to an arginine in APE2, is also labeled. (f) Structural alignment of the CCHC domain of ZCCHC4 and the CCHC zinc fingers of DHHC20 palmitoyltransferase (PDB 6BML). The rest of the DHHC enzyme is shown in transparent view. (g) (top panel) EMSA analysis of the 30-mer fragment of 28S rRNA titrated with increasing concentration of the ZCCHC fragments, visualized by SYBR Gold staining. (bottom panel) Coomassie blue staining of the same gel used for the EMSA experiment. Source data are provided as a Source Data file.

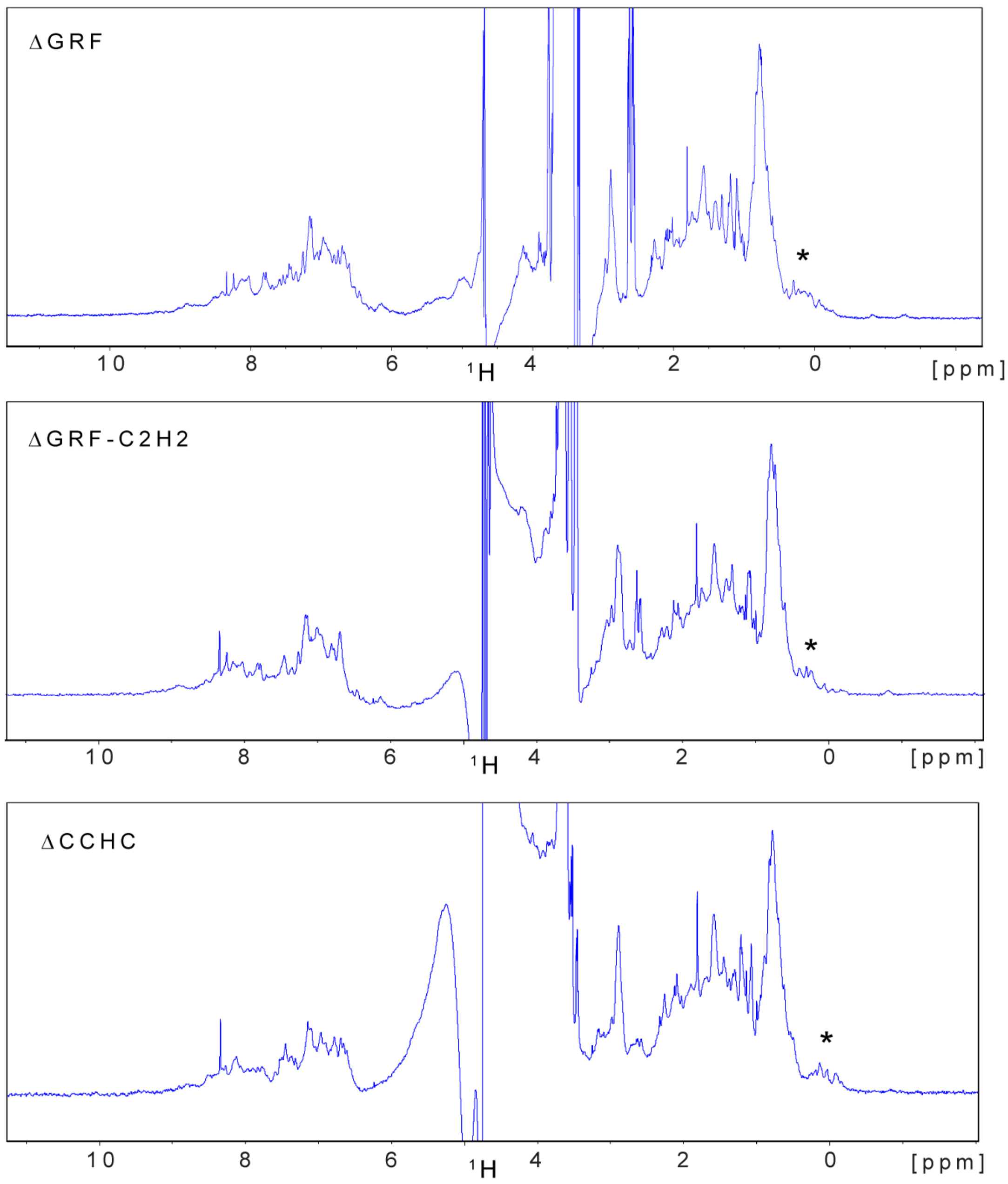

Supplementary Figure 6. ^{1}H NMR spectra of ZCCHC4 domain-truncated fragments. The low-frequency ^{1}H NMR signals, indicative of protein foldness, are marked by asterisks.

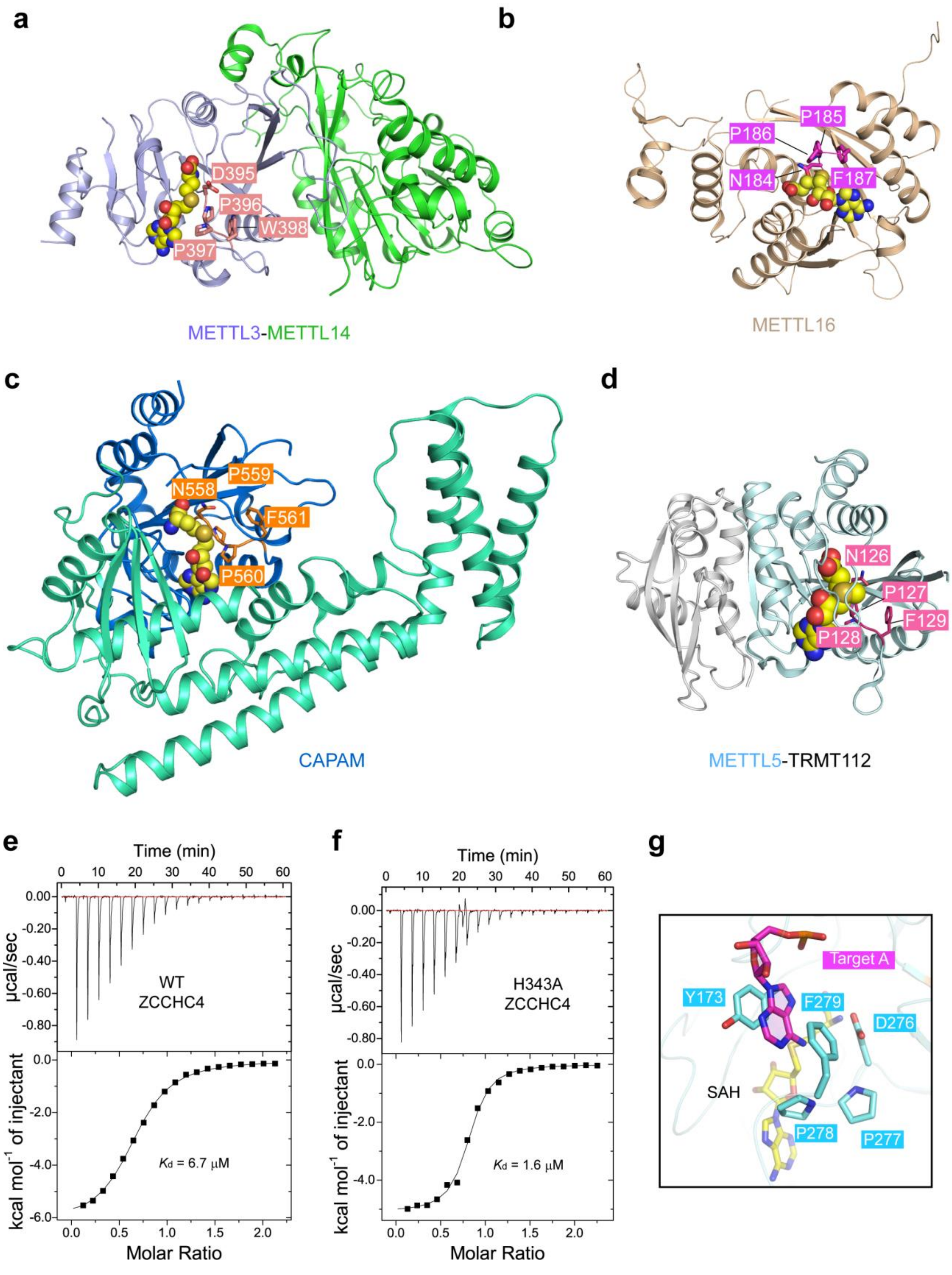
a
D395
P396
W398
P397
METTL3-METTL14
b
P185
P186
N184
F187
METTL16
c
P559
N558
F561
P560
CAPAM
d
N126
P127
F129
P128
METTL5-TRMT112
e
Time (min)
µcal/sec
WT
ZCCHC4
kcal mol-1 of injectant
Kd = 6.7 µM
Molar Ratio
f
Time (min)
µcal/sec
H343A
ZCCHC4
kcal mol-1 of injectant
Kd = 1.6 µM
Molar Ratio
g
Target A
Y173
F279
D276
SAH
P278
P277

Supplementary Figure 7. The active-site environment of ZCCHC4 is distinct from that of other m^{6}A RNA methyltransferases. (a-d) Ribbon representation of the METTL3-METTL14 (PDB 5IL2) (a), METTL16 (PDB 6B92) (b), CAPAM (PDB 6IRY) (c) and METTL5-TRMT112 (d) (PDB 6H2U) complexes. The side chains of the catalytic motifs of each complex are shown in stick representation. (e-f) ITC binding analysis of the SAM-binding affinity of WT (e) and H343A (f) ZCCHC4$_{24\text{-}513}$. Each ITC experiment was performed twice. (g) Structural model of the interaction between the active site of ZCCHC4 and target adenosine. The side chains of ZCCHC4 Y173 an F279 were slightly adjusted to accommodate the target base.

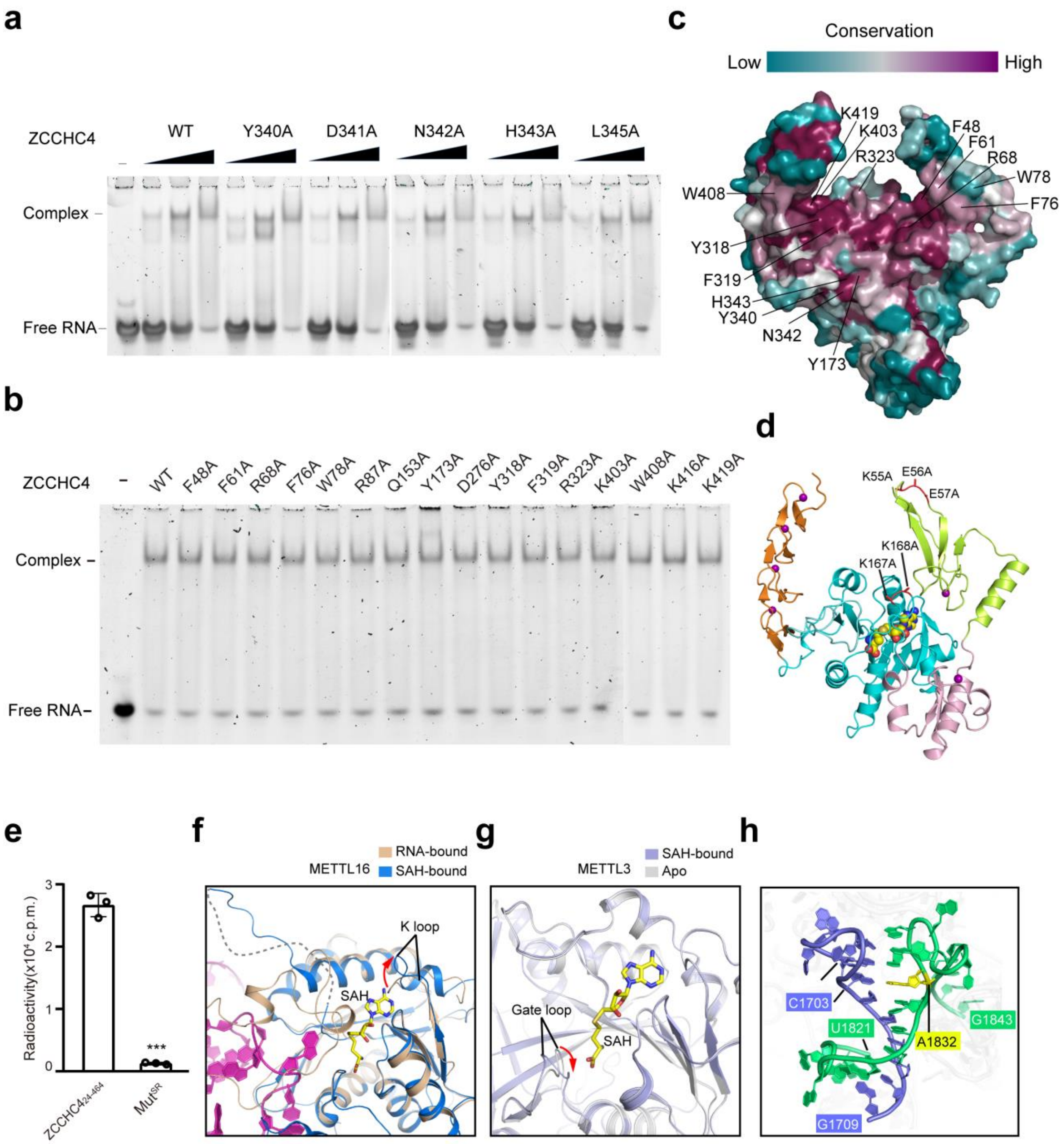

Supplementary Figure 8. Biochemical and structural analysis of the ZCCHC4-RNA interaction. (a) EMSA analysis of the binding affinity of ZCCHC4$_{24\text{-}513}$, WT or regulatory-loop mutants, for the 30-mer 28S rRNA fragment. (b) EMSA analysis of the binding affinity of 0.3 μM ZCCHC4$_{24\text{-}513}$, WT or mutants across different domains, for 0.6 μM 30-mer 28S rRNA fragment. (c) Sequence conservation analysis of ZCCHC4$_{24\text{-}464}$, interaction using the ConSurf server[5]. Residue D276, which was buried under the

surface, was not marked. (d) Ribbon representation of $ZCCHC4_{24\text{-}464}$, with the mutations (red) introduced for crystallization labeled. The mutation sites are colored in red. Otherwise the color scheme is the same as that for Figure 1b. (e) Methylation activities of $ZCCHC4_{24\text{-}464}$, WT or the surface-entropy-reduction mutant K55A/E56A/E57A/K167A/K168A (Mut^{SR}), on the 30-mer 28S rRNA fragment. ***, $p < 0.001$. Data are mean ± SD. (f) Structural comparison the cofactor-binding pocket of METTL16 in the presence of RNA (PDB 6DU4) or SAH (PDB 6B92). The conformational change of the so-called “K loop” is indicated by red arrow. (g) Structural comparison of the cofactor-binding pocket of METTL3 in the absence (PDB 5K7M) of presence of SAH (PDB 5K7W). The conformational change of the so-called “gate loop” is indicated by red arrow. (h) The cryoEM structure 18S rRNA (PDB 4UG0) highlighting the local structure of the methylation site Ade1832. The Ade1832 (yellow)-containing strand (green) is paired with another strand (slate) distant in sequence. Source data in (a), (b) and (e) are provided as a Source Data file.

Supplementary Table 1. Data collection and refinement statistics of ZCCHC4.

	PDB ID: 6UCA
**Data collection**	
Space group	$P\ 2_1 2_1 2$
Cell dimensions	
a, b, c (Å)	136.6, 290.4, 83.5
α, β, γ (°)	90, 90, 90
Resolution (Å)	50-3.10 (3.21-3.10) *
R_{merge}	0.126 (0.747)
$I / \sigma I$	10.6 (2.2)
$CC_{1/2}$	0.996 (0.922)
Completeness (%)	99.3 (96.5)
Redundancy	5.7 (5.5)
**Refinement**	
Resolution (Å)	47.68-3.10
No. reflections	60684
R_{work} / R_{free} (%)	24.9/28.2
No. atoms	
Protein	18467
SAH	156
Zn^{2+}	36
B factors (Å^2)	
Protein	105.4
SAH	76.9
Zn^{2+}	148.7
r.m.s. deviations	
Bond lengths (Å)	0.004
Bond angles (°)	1.05

*Values in parentheses are for highest-resolution shell.

Supplementary References

1. Akichika, S. *et al.* Cap-specific terminal N (6)-methylation of RNA by an RNA polymerase II-associated methyltransferase. *Science* **363** (2019).
2. Sledz, P. & Jinek, M. Structural insights into the molecular mechanism of the m(6)A writer complex. *eLife* **5** (2016).
3. Ruszkowska, A., Ruszkowski, M., Dauter, Z. & Brown, J.A. Structural insights into the RNA methyltransferase domain of METTL16. *Scientific reports* **8**, 5311 (2018).
4. van Tran, N. *et al.* The human 18S rRNA m6A methyltransferase METTL5 is stabilized by TRMT112. *Nucleic Acids Res* (2019).
5. Ashkenazy, H. *et al.* ConSurf 2016: an improved methodology to estimate and visualize evolutionary conservation in macromolecules. *Nucleic Acids Res* **44**, W344-350 (2016).
